# Evaluating a Psychological First Aid Training Intervention (Preparing Me) to Support the Mental Health and Wellbeing of Chinese Healthcare Workers During Healthcare Emergencies: Protocol for a Randomized Controlled Feasibility Trial

**DOI:** 10.3389/fpsyt.2021.809679

**Published:** 2022-01-27

**Authors:** Ling Wang, Ian Norman, Tao Xiao, Yamin Li, Xizhao Li, Mary Leamy

**Affiliations:** ^1^Clinical Nursing Teaching and Research Section, The Second Xiangya Hospital, Central South University, Changsha, China; ^2^Florence Nightingale Faculty of Nursing, Midwifery & Palliative Care, King's College London, London, United Kingdom; ^3^Department of Orthopaedics, The Second Xiangya Hospital, Central South University, Changsha, China

**Keywords:** mental health promotion, healthcare workers, psychological first aid training, educational intervention, crisis, randomized controlled feasibility trial protocol

## Abstract

**Aims/Background:**

The mental health challenges faced by frontline healthcare workers responding to emergencies have become a prominent public concern. Despite the consensus that Psychological First Aid (PFA) training can effectively support public mental health during emergencies through reducing acute distress and improving self-efficacy, yet it is concerning that previous flexible delivery and neglect for evaluating PFA training has resulted in unintended potential harms which may prevent further proactive uptake of this mental health prevention strategies. Establishing the feasibility of the PFA training through adapting to the local culture, tailoring to frontline healthcare context, and evaluating systematically may be helpful to inform a large trial, or ensure effective and sustained training delivery. This study aims to present a protocol for evaluating the feasibility and acceptability of a well-adapted PFA training intervention (Preparing Me) to address the implementation gap in this mental health promotion approach.

**Method:**

This is a two-armed feasibility randomized controlled trial (RCT) to be conducted among 80 Chinese frontline healthcare workers without prior related mental health training. Participants from the intervention group will receive an adapted PFA training program tailored to the Chinese frontline context to improve their knowledge and skills to support people in crisis. The primary objectives are to evaluate the training intervention's feasibility and the target population's acceptance of this educational intervention. The secondary objective is to obtain preliminary estimates of variability in participants' outcomes over a 3-months period. Measurements are taken pre-intervention (T0), post-intervention (T1), and at 1- and 3-months follow-up (T2–T3). A process evaluation using qualitative research with a subgroup of trainees, their clinical managers as well as trainers will be conducted to gain a comprehensive understanding of the intervention's acceptability and feasibility.

**Discussion:**

This present study protocol will help to establish whether this adapted PFA training intervention is feasible and accepted by the frontline healthcare workers, in preparation for a later effectiveness trial. It is anticipated that the resulted information would be an impetus to maximize usability and acceptance of this low-intensity PFA skillset by a wider population, thus supporting the mental health of frontline healthcare workers in dealing with crises for future emergencies.

**Trial Registration:**

This trial has been approved by the Institution Review Board from Central South University (LYG2020029) and by the Psychiatry, Nursing and Midwifery Research Ethics Committee at King's College London, England (LRS/DP-21/22-23161). It also has been processing registration at the Chinese Clinical Trial Registry.

## Introduction

The mental health challenges faced by healthcare workers is a major public health concern threatening health and economic prosperity of leading economies (1, 2). Working in crisis healthcare environments, healthcare workers providing direct services to patients have been experiencing increased patient volume and mounting demands, against a backdrop of limited resources; factors which are significantly associated with increased psychological stress (3, 4). This can in turn lead to burnout (BO), depression, anxiety disorders, sleeping disorders, and other mental illness (5). Health services' responding to the COVID-19 pandemic has been further exacerbated by these issues among frontline healthcare workers across the globe, which can, in turn, have a negative impact on health systems through decreased professionalism, and reduced care delivery and efficiency (6, 7).

The adverse mental health impact of public health crises on healthcare workers is not a new concern and is considered by some as a consequence of an historical underinvestment in mental health prevention for healthcare workers (8). There are two main issues here. Firstly, providing early psychological support to acutely distressed individuals has been well-recognized as critical to prevent the onset of mental illness, but the availability of early support is limited by insufficient onsite mental health professionals, limited resources, and insufficient recognition of the value of prompt psychological support for patients by frontline health workers themselves (7, 9). Secondly, apart from their pivotal role of caring patients, healthcare workers are at high-risk themselves arising from overwhelming stressful situations. Many are trained to prioritize physical problems rather than their own psychological needs—putting patients first; and acting as “heroes” whilst being conscious of the stigma associated with seeking emotional help for their own mental health distress (10, 11). The importance of individual healthcare workers recognizing and managing their own stress seems to be neglected.

Prior evidence has reported on short-term “mood boosters” to support the frontline workforce such as free-lunch, clapping for heroes, or practice telemedicine mindfulness (12, 13). Although these may offer temporary relief, they are no substitute for interventions to address the serious challenges frontline workers face regarding protecting their mental health and well-being. Some further argue that Self-Help Plus and stress management approaches may be ideal for stress and BO reduction (14), yet those appears to be insufficient of empowering them to address patients' need. Thus, investing in and accelerating preventive measures to reduce the workload burden on the healthcare workforce and practice self-care on a more permanent basis is imperative in the long term.

A recent scoping review of the impact of the COVID-19 pandemic on the physical and mental health of healthcare workers highlights the importance of fostering their resilience through proactive optimal provision of education, resilience training, and interventions to increase the confidence of staff in their preparedness to manage health care emergencies (7). Psychological First Aid (PFA) is one such intervention which has potential to deliver these benefits for healthcare staff capable of providing the necessary psychological support and practicing self-care. However, substantial challenges exist in its implementation may prevent its potential of this so-called cost-effective mental health promotion strategy.

### The Role of PFA to Support Public Mental Health During Emergencies

Psychological First Aid is an intervention to mitigate acute stress and foster resilience for crisis-affected victims which is promoted by public health authorities. Originally developed as a “do no harm” intervention to ensure the psychological recovery of people who have experienced traumatic stress in aftermath of the disaster (15, 16), PFA is effective to: (A) recognize their vulnerability (e.g., risk for crisis harm) and develop safety plans; (B) calm those who are emotionally distressed by the crisis; (C) improve disaster victims' self-efficacy to meet their emotional and practical needs; (D) support connectiveness and hope to protect individual well-being; and (E) facilitate access to more intensive assessment and intervention when indicated (16, 17).

Psychological First Aid as an early response intervention on reducing acute distress has several potential advantages. First, unlike traditional psychiatric interventions, PFA does not pathologize acutely distressed people; rather, it normalizes distress reactions and improves the coping abilities of traumatized disaster victims. This potentially reduce the stigma of being problematic in coping with crisis (18). Second, PFA does not involve psychiatric diagnosis; rather it involves approaching people in crisis, active listening, and providing practical assistance if needed via problem-solving, meeting basic needs or referring to professional care (19). Thus, it increases the scalability by training “lay people” to address the shortage of mental health professionals in frontline and reduce the stigma of seeking emotional help among acutely distressed individuals.

With the public emergencies increasingly prevailing across the globe and the emphasis of early psychosocial support in emergencies, PFA training has been widely applied as a first-line capacity-building approach to equip frontline workers within emergency response setting. A few studies conducted with frontline workers in humanitarian, disaster relief, and primary health sectors, have found that training in PFA has positively improved workers' knowledge of appropriate psychosocial responses and skills to support people in crisis situations, and enhanced their resilience to address adversity (20–23). Meanwhile, during the COVID-19 pandemic response, several institutions have started peer support programs of training in PFA for the support of healthcare workers. These reported anecdotal experiences suggest that it is a useful in-person, on-site support programme to foster resilience and self-efficacy (24–26).

The accessibility and utilization of PFA training to prepare especially the healthcare staff to ensure frontline psychosocial response is demanding, yet barriers to extend or proactively uptake PFA training to a wider group are substantial. First, the focus of PFA training on staff working exclusively in disaster settings which may lead to a misconception of PFA as a specific disaster response tool with less applications across non-disaster contexts and populations (27, 28). Second, most PFA training delivery is still the result of disaster-caused emergencies plans which result in just-in-time training. Current training delivery highlights the shortcomings of PFA training including inadequate guidance on adaptation, little motivation to evaluate, unclear training outcomes, and a lack of clarity about the core components of PFA training to ensure high fidelity (29). This is concerning that the previous flexible delivery with little motivation for evaluating PFA training has resulted in unintended potential harm to disaster victims, such as making false promises and confusion about role boundaries (30). Even worse, given the availability of evidence from training implementation science could further influence the therapeutic effect of the PFA intervention. Third, studies on the mechanisms through which PFA skills work to deliver patient benefit is still lacking, that may become the inhibitor to proactive scaling-up of training initiatives; for instance, few studies have investigated the longitudinal impact of PFA educational and implementation outcomes (e.g., trainees' adherence, preparedness, skills in real-world, well-being benefits).

Scholars suggest that PFA training should be accessible as rudimentary skill to increase frontline health care workers' coping ability, reduce their risk of distress and provide them with skills to deliver person-centered care (31). However, before establishing what extend potential benefits of PFA training can be contributed to the healthcare context, it seems to be neglected to determine the feasibility of the PFA training through adapting to the local culture, tailoring to frontline healthcare context, and evaluating systematically. Considering the COVID-19 pandemic is likely accelerate the uptake of PFA training, it is far more urgent to prioritize a well-adapted PFA training intervention thereby to effectively inform a randomized controlled trial (RCT) in a larger sample.

### “Preparing Me” Program—Support the Mental Health of Frontline Healthcare Workforce

In China, an inadequate investment to safeguard the mental health and well-being of the healthcare workforce has been well-documented during the COVID-19 pandemic response (32). Chinese healthcare workers have been increasingly required to respond to mental health crises arising from the growing psychosocial support needs of an enormous population, increased incidence of public health emergencies, insufficient, and unevenly distributed mental health resources, poor mental health literacy, and severe stigma toward help-seeking (33–35). The prior study has reported a remaining underdeveloped crisis mental health preparedness of healthcare workers and little attention is paid to capacity building through mental health training in China (36, 37). As highlighted by the COVID-19, it is far more imperative to establish evidence of a frontline capacity building approach to empower Chinese healthcare workers with the ability of recognizing and responding to psychological distress. Thus, this “Preparing Me” project (hereafter referred to as Preparing Me for frontline) comes at the opportune time which aims to adapt, tailor, and evaluate PFA training to frontline healthcare workers in China for use.

Among the three most influential PFA models, each with different priorities are the World Health Organization PFA guide (Psychological First Aid: Guide for Field Workers) (38), the National Child Traumatic Stress Network PFA guide (Psychological First Aid: Field Operations Guide) (39), RAPID model (The Johns Hopkins Guide to Psychological First Aid) (19). Of these, the present study involves adapting the John Hopkins Guide to Psychological First Aid for implementation and evaluation in China. The John Hopkins Model has been selected, over the other two models, because: (A) a small therapeutic RAPID PFA effect of reducing acute stress has been established in two RCTs among individual and group samples (40, 41); (B) its structured stepwise model aligns with empirical validation regarding its knowledge, skills, and attitudes (KSA) format for PFA competency statements so providing a foundation to quantitatively evaluate acquisition of the learning objectives (42); (C) the findings from John Hopkins' PFA capacity-building initiatives reveal a potential value of proactive preparedness to equip their public health workforce with PFA competencies representing 14 preparedness and emergency response centers (43–45). Given its emphasis on meeting the needs of proactively preparing frontline healthcare workforce for future emergencies, we have obtained permission from the John Hopkins Press (19) and the author (Professor George Everly) to: (A) translate the RAPID PFA guide into Chinese; (B) adapt this training into Chinese frontline context; and (C) evaluate this adapted Chinese PFA training intervention for frontline healthcare workers.

An appropriate adaption of a psycho-educational intervention is essential to maximize the implementation fidelity and enhance program fit (46). Given evidence on proper PFA adaptation resulting in PFA practice dilution (30), therefore, the adaptation of this Chinese PFA training intervention had been systematically conducted in line with these frameworks, i.e., the ADAPT-ITT cultural adaptation framework combined with the Bernal framework (47, 48). The aspects of language, metaphors, content, concept, goals, methods, and the context, as well as environmental and psychological factors had been well-considered when culturally adapting this training. Throughout this process, stakeholder participation in its development was achieved through: (A) conducting individual interviews (*n* = 15) of healthcare staff to capture their perceived needs (e.g., case range of application context) and training preferences; and (B) conducting three focus groups of frontline healthcare workers (with a total of 33 participants); and (C) using semi-structured discussions with an expert steering group to ensure cultural appropriateness. Drawing on the data collected from these stakeholders, we have identified possible adaptations and refined the PFA training intervention to fit Chinese healthcare practice. Thus, the “Preparing Me” PFA training intervention has been well-tailored to the Chinese frontline healthcare setting (see Table 1 of detailed PFA training intervention content based on TIDieR checklist) (49). Along with the consultation with experts in simulation and clinical education, this adapted Chinese PFA training program include a brief didactic introduction, role-play, case-simulation, and associated learning tools following an interactive and practice-based curriculum design (50–52). These will be delivered over 1-day face-to-face simulation training course (7 h) and two follow-up practice supervision sessions (1 h each); with a focus on improving the trainers' knowledge, skills, and self-efficacy related to support people in acute stress. All six modules comprise didactic information on mental health response to critical incidents and PFA, and each is framed by common challenges in frontline practice to increase its salience and provide practical approaches to implementing PFA into frontline healthcare practice. Training sessions are standardized via the use of a structured trainers' guide including up to 20 trainees. Since prior evidence on 1-day PFA training resulting in inconsistent PFA practice (30), we have added the two follow-up sessions to further support knowledge acquisition and skills development into their clinical practice.

**Table 1:** “Preparing Me” for Chinese frontline healthcare PFA training intervention.

**Training session (length)**	**Description of element content**	**Learning objectives**	**Implementation of element**	**Supporting materials**
Session 1: Introduction (30 min) Trainer: Psychologist	• What is PFA? • Theoretical background of PFA: • terms and concepts of early psychosocial support in emergencies • Historical context of PFA and its application	• Increasing awareness of mental health crisis, the context of applying PFA knowledge and skills	Location: Classroom lecture Mechanism: Group discussion	Theoretical dialectic slides Training handbook
Session 2: Establishing rapport (75 min) Trainer: Psychologist	• Reflective listening • Establishing rapport with acutely distressed people	• Increasing reflective listening through a greater awareness of one's emotion. • Fostering the ability to be supportive of one's emotional needs • Approaching people with proper communication skills	Location: Classroom Lecture Mechanism: Cased-based simulation Guided debriefing Group reflection	Theoretical dialectic slides Training handbook Case highlights
Session 3: Assessment and periodization (75 min) Trainer: Psychologist	• Assessment people's needs through listening to their story • Help people to prioritize practical concerns	• Enhancing the ability of assessing and prioritizing people with obvious urgent basic needs	Location: Classroom Lecture Mechanism: Watch video Cased-based simulation Guided debriefing Group reflection	Fast checklist leaflet Training handbook Case highlights
Session 4: Intervention and Referral (75 min) Trainer: Psychologist	• Calm people via cognitive reframing, stress management, etc. • Instill hope and connect people with loved ones and social support • Encouragement with further resources • Follow-up	• Focusing on stress reduction • Improving emotional regulation and thus lowering stress levels known to have a negative impact • Supporting continued care with referral resources based on local circumstances	Location: Classroom Lecture Mechanism: Cased-based simulation Guided debriefing Group reflection	Fast checklist leaflet Training handbook Case highlights Referral resources
Session 5: Selfcare and wrap-up (75 min) Trainer: Psychologist	• Risk factors of being a supporter • Self-care strategy	• Promoting self-care and generate motivation for care for oneself	Location: Classroom Lecture Mechanism: Group discussion	Stress management resources
Supervision session 1 (45 min) Supervisor: Psychologist	• Reflect PFA practice within frontline context	• Reflect real-setting skill practice	Location: WeChat Cased-based learning Mechanism: Practice case counseling	Supervision Handbook Referral resources
Supervision session 2 (45 min) Supervisor: Psychologist	• Proper evaluate PFA delivery • Develop supporting and safety plan	• Review knowledge and skill acquisition	Location: WeChat Cased-based learning Mechanism: Practice case counseling	Supervision Handbook Referral resources

*The reporting of this training content has been mapped using TIDieR checklist including theory (why), material and procedures (what), training provider (who), mechanism of delivery and location (how and where), training schedule and intensity (when and how much), adaptation (tailoring), adherence and fidelity (how well)*.

Thus, as a prerequisite to conducting a main RCT to evaluate its effectiveness in a larger sample, this present study is designed with the overall aim to establish the feasibility of this Chinese PFA training intervention (“Preparing Me”) to inform future design and process. It is the second phase of an inter-linked project, with the interest in exploring the potential of a capacity building program in response to vulnerable frontline health workers combatting COVID-19. It is anticipated that the resulted information would be an impetus to maximize usability and acceptance of this low-intensity skillset by wider frontline population, thus achieving the empowerment of the frontline healthcare workforce in dealing with crises through which preparing them the ability to recognize and responding to psychological distress.

## Objectives

This feasibility study is undertaken in preparation for RCT to explore uncertainties and enable us to optimize the intervention or the conduct of the trial. Thus, the primary objective is to determine the feasibility of outcome data collection with this systematically adapted PFA training intervention to the target population and obtain preliminary estimates of variability in participants outcomes, which could inform future sample size estimation for an RCT. The secondary objective is to generate the acceptability of this training intervention. Meanwhile, a nested process evaluation using qualitative methods will be conducted from diverse perspective in parallel to extend the acceptability and feasibility for randomized trial, as well as provide a comprehensive contextual information about potential barriers and facilitators related to PFA training intervention implementation. All detailed study objectives can be found in Table 2.

**Table 2:** Study objectives: Feasibility and acceptability.

**Domains**	**Research questions**	**Methods**	**Participants**
**Primary objectives: Feasibility**			
1-1 Recruitment and retention	• Can numbers of frontline healthcare workers be adequately recruited, trained, and retained for outcome analyses?	• Process outputs: trainees (number available in wards, number at training, number at supervision sessions; number completing measurement tools and time 1, 2 and 3)	Trainees
1-2 Adherence	• How many participants attend training sessions/supervision sessions? • What are the reasons for failing to attend training and supervision sessions?	• Attendance record for training and supervision sessions • Observation fieldnotes of training and supervision sessions • Qualitative interviews with expert trainers, and trainees	Trainees, trainers
1-3 Feasibility of measurement tools	• Are the assessment tools acceptable to participants for collecting training outcomes? • Are the measurement tools sensitive to assess the changes?	• Measurement tools completion rate, time for completion, number of missing items • Qualitative interview with participants about appropriateness of outcomes measures	Trainees
1-4 Overtime changes	• Do healthcare trainees' knowledge, attitudes, skills, well-being change after training, and is this sustained at follow up?	• Outcome's assessment pre-and post- training, plus 1- and 3-months follow-up • Calculation with mean score and standard deviation over time	Trainees
**Secondary objectives: Acceptability**			
2-1 Program evaluation	• Did attending training and supervision lead to trainees' improvement in practice, if so, what were these? • What do trainees think about PFA training program and supervision sessions?	• Qualitative interviews with trainers, trainees, and clinical ward manager think about the PFA training (is the format acceptable, is there anything missing, likes and dislikes, barriers, and challenges to complete the training and supervision) • Observation fieldnotes from training and supervision sessions	• Trainer, trainees, clinical ward manager
• 2-2 Fidelity of training intervention	• Did the trainers deliver the training as intended?	• Analysis of observation fieldnotes and transcripts of training session video recordings. Researcher rated PFA training intervention using a fidelity assessment tool.	• Trainer

## Methods and Analysis

### Design

**Figure 1:**
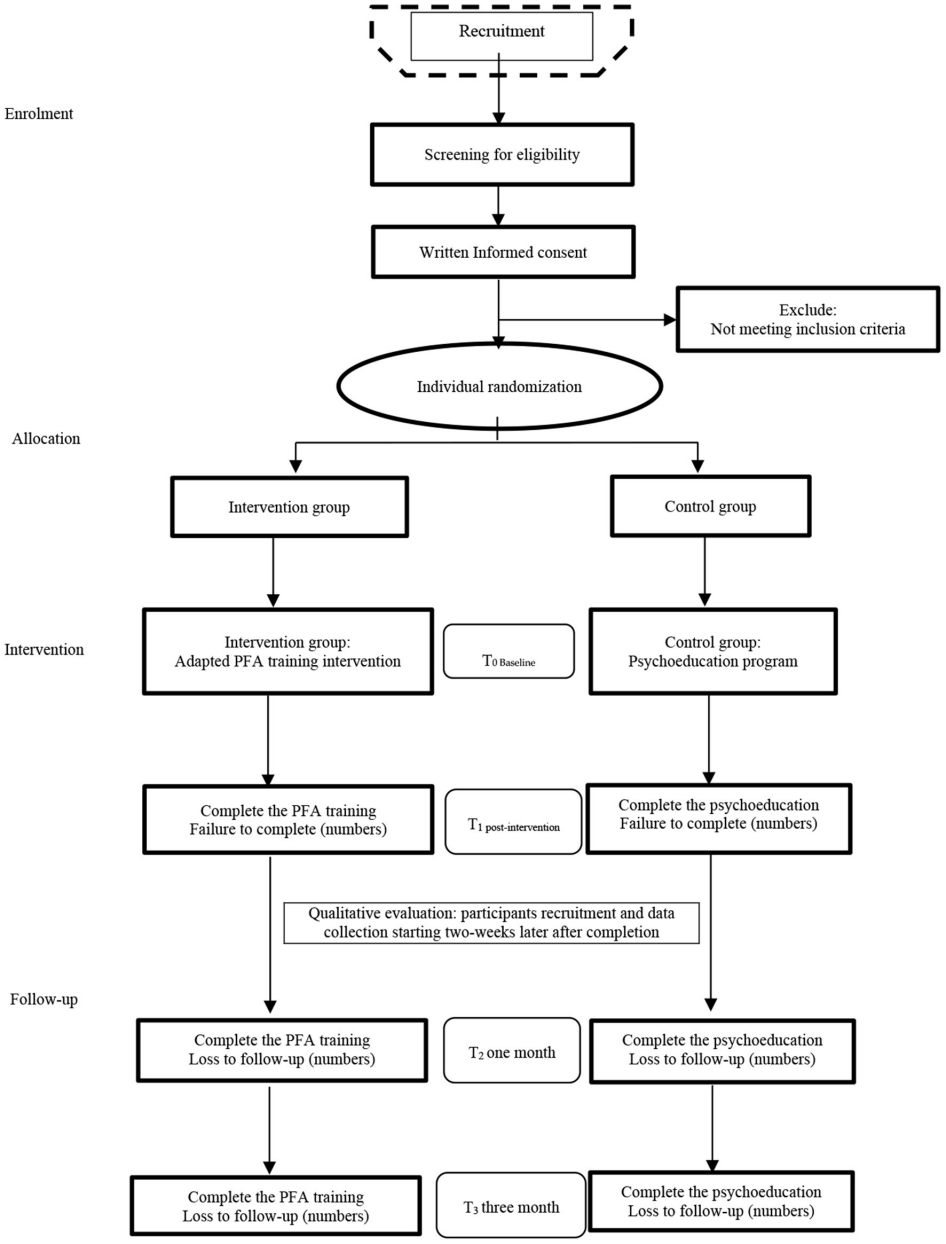
Study flowchart.

Given the implementation of PFA training intervention involves several components, including target a range of the behaviors, the trainers need mental health expertise and those receiving the intervention are experienced frontline healthcare workers, it can be described as a complex intervention. This study design has considered the Medical Research Council (MRC) framework, which is particularly suited to guide the adaptation and assessment of the feasibility/acceptability, with the intention of improving the design and evaluation, thereby to achieve an ultimately effective, sustainable, and scalable training intervention (53). Followed the guidance, this study protocol describes two steps of (A) feasibility trial evaluation following the recommendations of the SPIRIT 2013 statement [Standard Protocol Items: Recommendations for Interventional Trials] (54) and (B) process evaluation. The study flowchart is displayed in Figure 1.

#### (A) Feasibility Randomized Controlled Trial

A two-arm, feasibility randomized control trial will be used to test an adapted PFA training in a Chinese population of frontline healthcare workers. The purpose of the feasibility trial is to achieve two objectives (1) to determine the acceptability and feasibility of outcome data collection; and (2) to generate preliminary estimates of variability in trainee outcomes, which can inform sample size estimation for a large-scale RCT.

#### (B) Process Evaluation

Qualitative research will follow to add the feasibility and acceptability of this adapted PFA training intervention in a large tertiary hospital setting to complete interviews and observations with trainees as well as their clinical ward manager and trainers. Those generated potential contextual information about this feasibility RCT from multiple stakeholders will help to inform future intervention refinements and study design (55, 56).

### Feasibility Randomized Controlled Trial

#### Setting

The recruitment of participants and implementation of the “Preparing Me” PFA training intervention will be conducted in a representative Chinese tertiary hospital (The Second Xiangya Hospital of Central South University) in Hunan Province, the central part of China.

**Figure 2:**
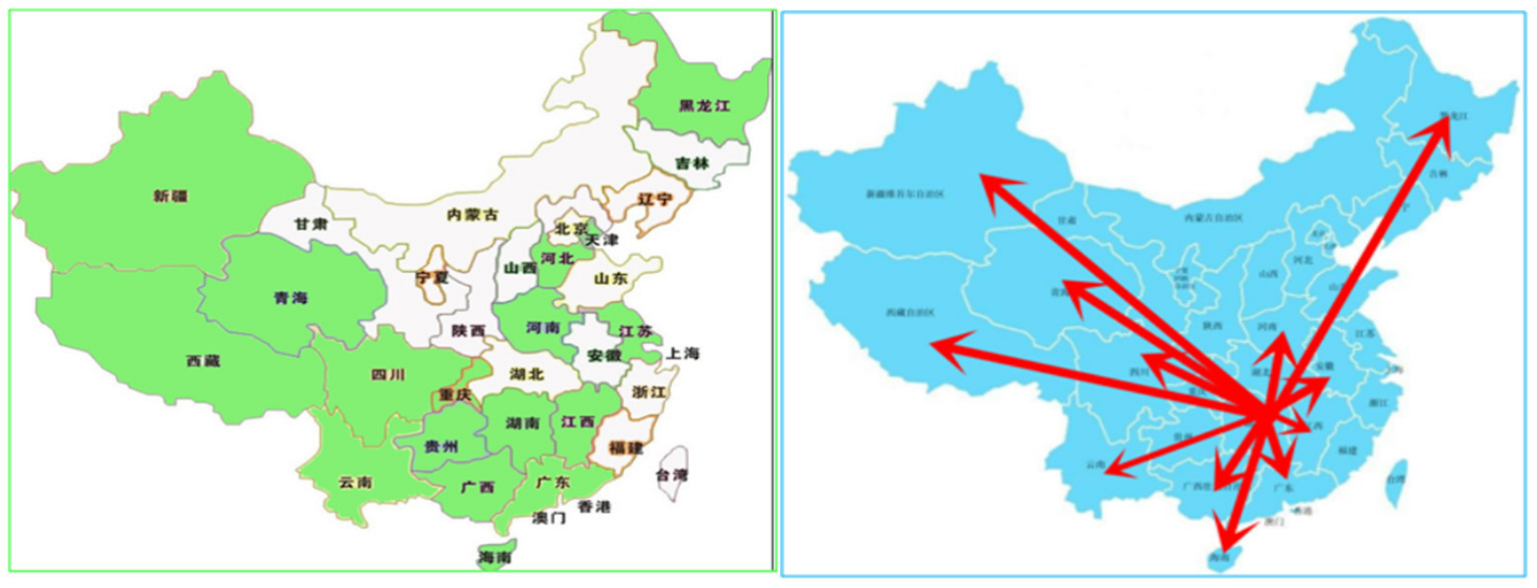
Response map—summary of selected national and regional emergency response by the Second Xiangya Hospital of Central South University.

It has been selected because of the following three characteristics: First, this is a designated emergency response hospital in China that they have established a national emergency medical rescue team which has been approved by the National Health Commission of the People's Republic of China in 2012. Within China's four-tier response system for public health emergencies that determines what measures a region will implement, with level I the most serious, every designated regional medical institute will take on the responsibility of emergency medical rescue accordingly. Generally, the approved national rescue team will be called out not just national major public health emergency but also regional rescue services. Therefore, as a representative emergency response center, healthcare workers working in this hospital are regularly serving the role of responding to natural disasters, infectious disease outbreaks, and other critical incidents at all levels. According to the hospital administration data, healthcare workers have been dispatched by national authorities to provide emergency rescue services on 80 different occasions for a range of natural, social security, and public health emergencies during the past 7 years. These include national major emergencies (e.g., Ya'an earthquake in 2013, Sinking of Dongfang zhi Xing in 2015, Tianjin Explosion in 2015, Wuhan first wave of COVID-19 pandemic in 2020) and regional emergencies (e.g., Yiyang Floods in 2018, Changde car accident in 2019, Leiyang coal mine flooding accident in 2020, Zhangjiajie 2nd wave of COVID-19 in 2021) (see the Response map in Figure 2 which summarize of selected national and regional emergency response by the Second Xiangya Hospital of Central South University). Second, it is a represented public emergency preparedness center in the Hunan Province which has developed a centralized response system, thereby instilling an emergency response literacy among their staff team. For example, their centralized working model shows great advantages that makes it contain the spread of COVID-19 effectively to support Wuhan city in 2020, e.g., their massive capacity and comprehensive functions, including “fever-clinics” to screen patients, a COVID-19 department to isolate patients, and many makeshift beds as emergency wards. Under several years of team construction and medical rescue training focus, their future preparedness efforts warrant a focus on improving their capacity-building, especially the mental health response and improving their own workforce's well-being. Third, it is a top-ranked tertiary hospital as well as a key national center for healthcare, education, and clinical research in China which has 3,500 beds, 40 clinical departments, and 127 ward areas facing complex healthcare service demands within their regular hospital services in central China which makes them explore the complicated challenges in their practice context. The national simulation centers for experimental teaching and training platform offers an opportunity to design and test this capacity-building project.

#### Trial Participants

This trial will sample frontline healthcare workers, who provide services directly to patients and are the first and often only link to essential health services. The sampling frame includes all professional disciplines of frontline healthcare workers, namely nurses, midwives, doctors, pharmacists, who are employed by the Second Xiangya Hospital of Central South University which is part of the national and regional emergency rescue service in China (i.e., they would be called to provide rescue services to individuals in the event of a disaster or public health emergency).

The eligibility inclusion criteria to participate in the trial are: (A) participants must be over 18 years old; (B) participants are employed in departments that would be called to emergency response (e.g., emergency departments, infectious disease, orthopedic, intensive care unit, etc.); (C) participants are non-mental health frontline healthcare workers. The exclusion criteria to participate in the trial are: (A) they say for whatever reason that they are unavailable to participate in the PFA training course for whatever reason; (B) those who have prior knowledge in related mental health areas (e.g., completed education in a mental health related field or similar training or delivery).

#### Sample Size

A sample size of between 24 and 50 is considered reasonable in a feasibility study, to be able to estimate a participation rate in anticipation of a full trial (57, 58). Informed by this, the target recruitment for this feasibility trial will be *N* = 80, allowing for a 20% loss to follow-up rate and for at least 64 participants to complete the study during the COVID-19. The sample size is projected to provide enough numbers to be able to estimate the likely efficacy and acceptability of the training intervention to inform the design of a future RCT.

#### Recruitment

Participants will be recruited from the Second Xiangya Hospital of Central South University through the following two ways: (A) Direct methods: access to research participants will be via two gatekeepers. The first gatekeeper will be the Director of the nursing department in the local hospital, who distribute the study information to the clinical departments managers on behalf of the researcher, (i.e., a communication message/talk based on the email/phone recruitment script). The second gatekeepers will be the clinical department managers who will help to identify the staff in service who are interested in participating and ask them to contact the researcher directly. (B) Indirect methods: Advertisement posters will be posted onto the hospital notice board and be disseminated via social media posts through individual WeChat moments. Participants who are interested in participating will be asked to contact the researchers via a dedicated email address, phone number, and WeChat message.

Once potential participants have expressed an interest in being involved, they will be assessed against the eligibility criteria by the researcher, and if they are eligible, they will then be asked if they have read the participant information sheet. Meanwhile, they will be given an opportunity to ask any questions, and if being addressed in satisfaction, they will be asked to give their written informed consent for participation in the trial. They will be asked to complete the baseline assessments and then randomization to either intervention or control group of the trial will occur.

#### Randomization

The randomization will be performed by an independent researcher not connected with either participant recruitment or data collection, using a 1:1 ratio as per a computer-generated randomization schedule using randomly permuted block sizes by randomization.com. All eligible participants who give written informed consent will be randomly allocated to either the intervention or control arm of the trial. Due to the nature of the study, only the researcher collecting/analyzing the data from trial participants can be masked to the randomization.

As a check on blindness, the researcher will be asked to guess the group allocation of trial participants after they have completed each final follow-up assessment. Unmasking will not occur until databases are closed and the main analysis has been completed.

### Intervention

#### Intervention

The whole process of this PFA training adaptation for delivery to Chinese frontline healthcare workers has been systematically described elsewhere. A detailed description of the Chinese ‘Preparing Me’ PFA training content has already been structured using the template for intervention description and replication checklist (TIDieR) in Table 1. Four trainers who have teaching and clinical psychological counseling background will be trained to deliver the PFA training intervention. Each of the six modules will be delivered in 75-min session blocks, incorporating didactic lectures, group exercises, role playing, and discussions to encourage active engagement and to improve the acquisition of PFA knowledge, skills, and techniques. All these sessions will be audio-recorded in preparation for the researchers to assess the fidelity using a PFA fidelity assessment tool. To support frontline healthcare participants to be able to apply the knowledge, skills, and techniques to their practice, two WeChat-based online supervision sessions will be offered.

#### Psychoeducation Programme (Control)

Participants in the control arm will receive the best available alternative to PFA training, which in this setting is the hospital's existing 1 day psychoeducation training course. This involves a series of lectures on basic mental health training topics, including the traditional Chinese culture, understanding psychological crisis and skills, referral resources, and ethics.

This study will attempt to minimize contamination between the two arms through (A) engaging different trainers with equivalent clinical and training experience to deliver the training; and (B) consenting participants in both arms will be asked not to share information regarding the training that they have received with colleagues who might be involved in a different arm of the trial.

### Primary Objectives

The primary objective (i.e., the acceptability and feasibility of outcome data collection) will be collected by the researcher (LW, XL). These include data on (A) recruitment and retention, (B) training adherence and (C) the feasibility of the outcome measures which describes as below:

(A) Recruitment and retention
  - Number of eligible participants approached and consenting to take part, which is randomized, as well as the number of ineligible participants
  - Number of participants who successfully complete training
  - Number of participants attending online supervision session/s
  - Number of participants lost due to follow-up and dropout rate
(B) Training attendance (adherence)
  - Number of sessions attended
  - Time dedicated to practice
  - Test after all session
(C) Feasibility of measurement tools
  - Time taken to fill in questionnaires
  - Missing data from questionnaires

### Secondary Objectives

The secondary objective (i.e., variability in participants outcomes) will collect two domains of measurement data that capture possible outcomes, mediator, moderator, or covariate to be included in any future effectiveness trial to quantitatively determine the acceptability and feasibility of randomized trial: (A) preparedness via knowledge, skills, and attitudes, and PFA practice in real setting; and (B) related psychological measures of resilience, self-efficacy, coping, post-event symptoms, post-traumatic growth, general health, and quality of life.

(A) Preparedness
  - *Knowledge, Skills, Attitude*. The PFA Knowledge, Skills, and Attitudes Survey Form is a self-report, five-point Likert scale composed of 25 items organized by knowledge (10 items), skills (7 items), and attitudes (8 items) used to evaluate the learning effect of the PFA training intervention. It has been adapted from the Johns Hopkins PFA training initiative evaluation study and is empirically supported (44).
  - *The PFA Training Follow-up Questionnaire*. It is an 11-item survey distributed to trainees twice, at 1- and 3-months after the training to determine the extent to which trainees have applied PFA knowledge and skills and their views on the acceptability and usefulness of PFA applied to persons who incurred crisis during a disaster event or other incident.
(B) Related psychological measures
  - *Brief Resilience Scale*. The Brief Resilience Scale consists of six items identifying one's ability to bounce back from stress developed by Smith {Smith, 2008 #15}. It is a five-point rating scale (1 = strongly disagree; 5 = strongly agree) and scored by reverse-coding Items 2, 4, and 6 and calculating the sum of all six items. This scale has established psychometric properties, exhibiting an acceptable internal consistency with Cronbach's α values equal to 0.76 and 0.72, respectively, in the Hong Kong and mainland samples (59, 60).
  - *General Self-Efficacy Scale*. The General Self-Efficacy Scale (GSE) has become a widely used instrument for measuring general self-efficacy (61). The scale consisted of 10 items and each item was scored on a four-point Likert scale from 1 (strongly disagree) to 4 (strongly agree). The Chinese version of this scale has showed good reliability and validity in Chinese population (12).
  - *The Impact of Event Scale-Revised*. The Impact of Event Scale-Revised will be used to determine the frequency of post-traumatic symptoms in relation to a recently experienced work-related traumatic event (62). It is a 22-item scale rated on a five-point Likert scale using anchors between 0 (not at all) to 4 (extremely) and total score range is 0–88 (higher score indicates greater severity). Good psychometric properties for the total scale and each subscale have been previously reported in Chinese sample (63).
  - *The Simplified Coping Style Questionnaire*. The Simplified Coping Style Questionnaire (SCSQ) is used to assess the participants' attitudes and actions individuals that would take in the face of the life events (64). It is a 20-item self-report four-point Likert scales (0 = never; 3 = very often) that includes two dimensions: active coping (12-item) and passive coping (8-item). The instrument has demonstrated that the test–retest reliability coefficient of this instrument is 0.89, and Cronbach's α coefficient is 0.90 in Chinese populations (65).
  - *Post-traumatic Growth Inventory*. The Posttraumatic Growth Inventory (PTGI) is developed to assess positive outcomes in response to traumatic events (66). The 21-item scale assesses positive psychological change in the wake of struggling with highly challenging event circumstances. Good psychometric properties of this instrument has been established in Chinese population (67).
  - *General Health Questionnaire-12*. The General Health Questionnaire-12 is an 12-item self-report checklist devised by Goldberg is one of the most widely applied assessments of the severity of symptoms associated with psychological distress (68). The questionnaire contains 12 items, each scored on a four-point Likert scale from 0 to 3. The Chinese version shows a Cronbach's α coefficient was 0.86 for the overall GHQ-12 (69).
  - *Professional Quality of Life*. The Professional Quality of Life Scale (ProQOL-5) is 10 items scale to assess one's perceived quality of life in relation to working as a helper and includes both the positive and negative aspects of this work (70). It is a five-point Likert self-reporting scale and consists of three components of professional QoL: compassion fatigue (CF), (BO, and compassion satisfaction (CS). The Cronbach's α coefficients for each dimension range from 0.758 to 0.821, and the test–retest reliability coefficients range from 0.764 to 0.867 (71).

### Data Collection

**Figure 3:**
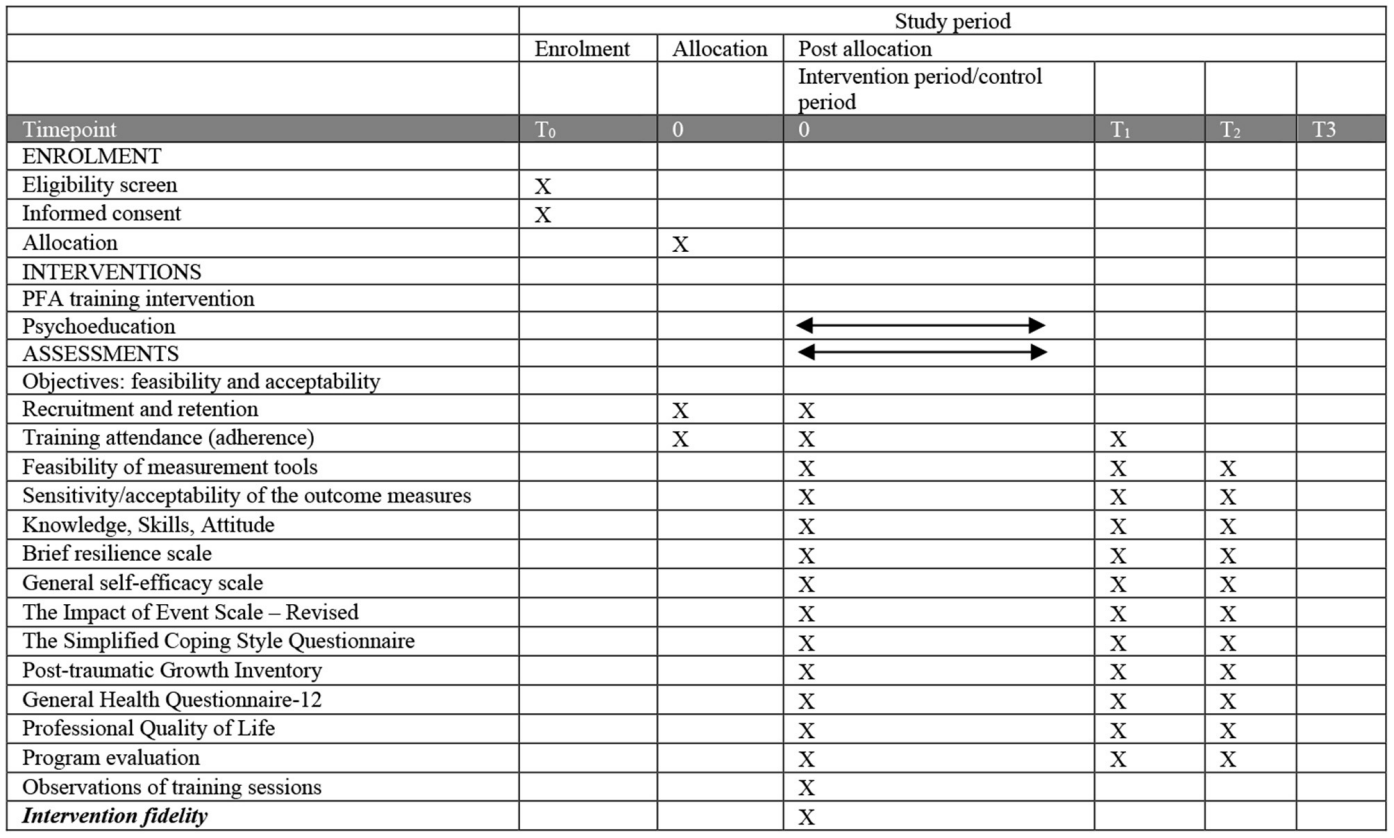
Schedule of enrolment, interventions, and assessments.

The schedule of enrolment, interventions, and assessments has been summarized in Figure 3. Demographic data will be recorded at baseline only, including age, gender, religion, marital status, living conditions, educational background, professional title, specialities, years of working experience, and experience of coping with crisis. Quantitative data will be collected from participants at three different times: baseline data will be collected before the PFA and psychoeducation training programmes (T0), the initial follow-up data will be collected immediately after training programmes are completion (T1), 1- and 3-months follow up data that will be followed up 1- and 3-months after the programmes are completed (T2, T3). The researcher will conduct data collection by providing each participant with a study pack at each time point. After presenting the information, the participants will be asked to complete these packs of assessment tools. To encourage participants' attendance at baseline and follow-up sessions, participants will be contacted by WeChat message at least 1 week before each data collection timepoint. The 1- and 3-months follow-up post-intervention measurement will be collected online via WeChat Wenjuanxing—a platform providing functions equivalent to Amazon Mechanical Turk. All questionnaires can be seen in Supplementary Materials.

### Statistical Data Analysis

Since this is a feasibility study and is not testing hypotheses regarding effectiveness of PFA training intervention, inferential statistics will not be employed. The primary objectives will be addressed via generating estimates of dispersion and central tendency (i.e., proportions, means, SDs, etc.). The acceptable levels of eligibility and enrolment will be a 90% or greater screening eligibility rate for frontline healthcare workers referred to the study. A recruitment rate is expected of 15 trainees per week and the target sample size would therefore be achieved in 6 weeks. The acceptable proportion of enrolled trainees who complete all the training sessions will be 80% or greater, given that there's 18% dropout rate of participants in PFA training cluster RCT (23). The feasibility of collecting quantitative outcome data includes (1) <20% missing data at the group level for each time point and (2) generating estimates of effect and variability related to by the independent research facilitator. Quantitative secondary objectives will be addressed via generating bivariate correlations and regression coefficients for the selected measures. Correlations between the baseline and follow-up will be estimated for continuous outcomes. Measures will be examined for multi-collinearity, as well as any needed transformation due to unfavorable skewness and kurtosis (72, 73). Appropriate 95% confidence intervals will be reported, with differences deemed significant at the 5% level.

### The Nested Process Evaluation

Research on using qualitative methods for feasibility RCT will be likely of particular value in refining understanding of how the intervention works and facilitating ongoing adaptation of intervention and evaluation design in preparation for a full trial (74). Thus, this study will nest a process evaluation, consisting of (A) qualitative interviews and (B) observations of the training sessions, with the aim to extend a more in-depth understanding of the acceptability and feasibility of PFA training intervention for this feasibility trial. First, individual interviews will be applied with trainees, as well as their clinical managers and trainers to reflect the perceived experience and impact of the PFA training, as well as barriers and facilitators to the training completion and program fit in. In particular, this study adds interview with participants' line managers from the clinical ward, whose role is to provide supervision of trainees practice after training completion, it is in consideration with lacking evidence in real practice from previous PFA training evaluation (29). Second, observations with trainees and trainers in the classroom will be used to expand the immediate response and feedbacks for the training, and to assess the level of intervention fidelity.

#### Sample

The process evaluation sample will use purposive sampling strategies in order to triangulate findings (75). Participants who will be invited to be interviewed include three different participant groups, including (1) trainees (*n* = 12) are those attending all the training sessions; (2) trainers (*n* = 4) who have been involved in this training; (3) clinical ward managers (*n* = 5) from PFA trainers' clinical ward. Maximum variation sampling will be considered via inviting trainees and managers representing diverse levels/types of training to participate and mangers in diverse working context to obtain rich understanding (76). Given the nature of the target qualitative sample participants, we expect this sample size will achieve data sufficiency.

#### Recruitment

Trainees who receive PFA training sessions and practice PFA after training completion in frontline will be sent an invitation for the interview via WeChat messages, and if they agree, their clinical manager will then be invited. The trainer of the PFA training intervention group will be invited to participate by the researcher prior to the start of the training and supervision sessions.

#### Data Collection

##### Interviews

After written informed consent is obtained, qualitative interview with trainees will be conducted at 2 weeks after the training completion, while interview with trainers will be conducted after they have delivered the training and supervision. An indicated topic guide (see Table 3) is informed by the objectives of feasibility and acceptability which will guide the qualitative data collection. Interviews will take place on hospital premises, and the length of time taken range from 30 to 40 min.

**Table 3:** Indicative topic guide after the PFA training completion.

**Trainees**	
Acceptability and feasibility	How would you describe your experience of completing this training program? How do you value the PFA in your clinical practice? Any indicative changes in behaviors? How would you describe your experience of supporting your patients or colleagues through providing them PFA skills?
Satisfaction	How satisfied are you overall with training module? How clear do you find the concepts and ideas in the module?
Suggestions for further improvement	What do you think could be improved about? Is there anything missing?
Barriers	Are there any difficulties to taking part? What are the barriers to completing training course, and applying training to practice? What are the challenges to complete the assessment, with questions relating to reasons for not taking part/ discontinuation or dropping out?
Process of change	Are there any changes in your perspective of coping acute distress? If the answer is “Yes”, what are they?
**Trainers**	
Acceptability and feasibility	What they thought about the training, what worked, what didn't work?
Satisfaction	How satisfied are you overall with the training session adaptation and development? How well do you find the concepts and ideas organized in the module?
Suggestions for further improvement	What do you least like about the program? What do you think could be improved about? Is there anything missing?
Barriers and facilitators	Are there any difficulties to deliver the training? What are the barriers to deliver training course? What are the challenges to improve training refinement?
**Clinical ward managers**	
Acceptability and feasibility	What is your perspective on the value of psychological first aid for supporting your staff's work? Can you talk about your experience of supporting your staff to participate this training?
Satisfaction	How satisfied of your staff's practice after training completion?
Suggestions for further improvement	What do you least like about the program? What do you think could be improved about? Is there anything missing?
Barriers and facilitators	Are there any difficulties to make them participate the training and practice PFA on frontline? What kind of support would need to enable your team's participation in this training opportunity?
Process of change	Are there any changes in your perspective of your staff to responding to acute distress? What important aspects of diversity you feel when you take a view on this psychological first aid in clinical practice?

##### Observations of Training Sessions

The PFA training adaptation the training sessions will be recorded so the researcher can observe the sessions afterwards. Researchers will make fieldnotes about the delivery of and response to the sessions provided on the program, barriers of training completion, and trainers of task engagement and on its overall structure, delivery, and content of the training (see Table 4). Also, the fidelity of the adapted Chinese PFA training programme will be evaluated. Fidelity checklists will be created for each of module session types to measure how closely they adhere to the RAPID PFA model manual and the intended aims of the interventions based on the domains provided by the Health Behavior Change Competency Framework (HBCC) (77). All training and supervision sessions will be video-recorded and watched by the researcher to rate against the fidelity checklists to ensure training and supervision are delivered as intended (see Table 5). Field notes completed by the trainers during the supervision will also be documented to evaluate whether the participants follow the actions of PFA which may be relevant to the study analysis.

**Table 4:** Observation checklist of PFA training delivery.

To what standard are the core content being delivered?
Are the standard manual and resources being used?
To what extent are the key learning outcomes of each session achieved?
Rate the facilitator 1) Variety of approach to each session using facilitation, presentation, coaching, activities 2) Engagement/rapport with group 3) Group management/involvement 4) Challenge negative behavior within group 5) Positively addressed resistance to program
Additional comments:

**Table 5:** Fidelity checklist of PFA training delivery.

**Domain**	**Assessment method**	**Quality criteria**
Training intervention design	• Assess whether PFA training manual reflect the underlying theoretical model	Prior to study implementation, investigators, and optimally a protocol advisory, should review PFA training manual to ensure that the active ingredients of the RAPID PFA model are fully operationalized. The degree to which the measures reflect the hypothesized theoretical constructs and mechanisms of action should be assessed.
Trainers	• Assess trainers' skills acquisition	Ensure trainers are trained to a well-defined performance Trainers' role-plays should be evaluated for both adherence to PFA components and adherence to process (e.g., interactional style)
Receipt of all PFA training sessions	• Assess degree to what extent of participants understood intervention • Assess participants ability to perform the skills	Verify the participants' understanding of the PFA training information provided Verify that they can use the skills and recommendations discussed (via pre–post-tests, recordings).

### Qualitative Data Analysis

Transcripts of interviews with trainees, managers and trainers, as well as field notes will be imported into NVivo software for data management and analysis using direct content analysis; thematic analysis and summative content analysis (78–80). This combined method focuses on developing common themes that are represented in the data and using open coding of a selection of transcripts, initial key themes, and emerging patterns will be identified, thus enabling development of a coding frame which will be refined and applied to the full dataset. Direct content analysis will establish a codebook with a set of a codes corresponding to the acceptability and feasibility objectives, while using the reflective thematic analysis and analytical memoing would expand the codebook through identifying new concepts, constructs, and factors. To further quantify the number and salience of finalized codes within and across interviews from the data sources, summative content analysis, and constant comparison will be conducted. The initial code book will be created by the researcher LW and be validated by the team members discussion with expertise in qualitative research methods, clinical education, and mental health science to achieve a consensus-based codebook. The analysis will be conducted in the original Chinese language, and final themes will be translated into the English language by two researchers that could ensure the accuracy and transparency of the data.

### Data Monitoring

Data quality is ensured through several mechanisms, including referential data rules, valid values, range checks, and consistency checks. Checks are applied at the time of data entry into a specific field and monitored by a central data safety and monitoring group.

### Adverse Events Reporting and Harms

The main risk factor is psychological distress among trainees during the training sessions simulation or practice. A contingency plan regarding the use of videotaped testimonials and provision of a stand-by counselor to provide immediate care to the trainees when required has already developed which is explained in the participant information sheet. All changes resulting from adverse events or serious adverse events will be documented. Any participants who become distressed or require additional support during the study's training will stop the training and the person will be invited to a private room and provide a leaflet with information about appropriate sources of support they can access. The trainer who can provide on-site counseling will be able to respond to their needs. If the participant needs a further referral, e.g., medication, or treatment, the researcher will help to receive mental health care and contact.

### Ethical and Dissemination

This trial has been approved by the Institution Review Board from Central South University (XTXL20200610) and by the Psychiatry, Nursing and Midwifery Research Ethics Committee at King's College London, England (LRS/DP-21/22-23161). It also has been processing registration at the Chinese Clinical Trial Registry. Also, this project has been supervised by the PhD researcher's supervisors who are experienced in mental health research. All participants will be asked to provide their written and informed consent acknowledged that all identifying information will be removed.

Several key deliverable and knowledge dissemination strategies will be employed. Findings will be summarized to submit to open-access journals and results will be shared broadly with the policy, practice and academic communities via conference attending and seminars. The use of digital and social medic will be adopted to disseminates project updates and results, as well as generate brief, professionally produced infographics and summaries for dissemination.

## Discussion

Prioritizing the mental health and well-being of healthcare workers is considered an urgent global public health priority (6). Training in PFA has been considered effective to support public mental health in emergencies through reducing acute distress and improving self-efficacy, public health authorities disseminate the training across the globe with the expectation to enhance population level of mental health preparedness and resilience in emergencies. However, a flexible delivery with a neglect for evaluating PFA training has resulted in unintended potential harms, and further prevent generating large effects, which may hamper future proactive uptake and achieve a large level of preparedness, especially for the frontline healthcare workforce. Before establishing what extend potential benefits of PFA training can be contributed to the healthcare context, it has been neglected that determining the feasibility of the PFA training through adapting to the local culture, tailoring to frontline healthcare context, and evaluating systematically is crucial. Considering the COVID-19 pandemic is likely accelerate the uptake of PFA training, it is far more urgent to determine the feasibility of a well-adapted PFA training intervention thereby to effectively inform a RCT in a larger sample. Thus, this study protocol presents a randomized controlled feasibility trial protocol to determine the feasibility and acceptability of a well-adapted PFA training programme for Chinese frontline healthcare workers to improve their ability to provide early emotional support to those in need and self-care, especially in relation to responding to public health emergency situations.

This study has several strengths. First, to our knowledge, this is the first rigorous evaluation of a systematically adapted PFA training intervention for use by frontline healthcare workers which could help to decide the larger follow-on studies of this low-intensity capacity-building programme. Second, as a feasibility-controlled trial combined with a process evaluation, the combination of data regarding retention rate, the ability of the training program to lead to changes in the secondary objectives of interest (i.e., increased preparedness and self-efficacy, reduced burn-out, and secondary traumatic stress, etc.) gained will shape the design and inform a sample size calculation for a future trial. Experience from the training practice will generate a rich and comprehensive understanding of how PFA training might be delivered in the context of a frontline health service and so potentially widen its application to Chinese population. Third, considering that the sample setting has the combing role of emergency response and regular health service in frontline healthcare context, it may contribute to enhance the understanding of its relevance and applicability of findings in different regions, especially in non-disaster frontline settings. Given the overall aim of developing preventive strategies to improve mental health crisis preparedness for frontline healthcare workers, it is consistent with objectives of addressing frontline challenge in public mental health during complex emergencies (81).

## Limitations

There are some limitations of this project. First, the design and evaluation of the feasibility trial has predominately focused on the outcomes associated with the frontline healthcare workers rather than the outcomes for recipients of PFA following a disaster, public health emergency, or other crisis. Second, the timeframe for follow up outcome measures is only 3 months, which may be insufficient time for PFA trainees to apply their knowledge and skills in practice evaluation.

## Conclusions

Psychological First Aid training has been widely proposed as a mental health prevention approach of providing early support to acutely distressed individuals and improving self-efficacy of frontline healthcare workers in responding to crisis, yet it has not been fully evaluated which result in significant flaws of its implementation. This study protocol is designed to evaluate the feasibility and acceptability of a well-adapted PFA training intervention (Preparing Me) for future effectiveness assessment. If it could achieve an 80% retention rate in the end, a main RCT of this educational intervention will be decided, expecting the overall aim of promoting their capacity of recognizing and managing stress when confronted with healthcare emergencies, with an expectation to boost evaluation effort to improve this prevention training approach to achieve a population preparedness. It is anticipated that the resulted information would be an impetus to maximize usability and acceptance of this low-intensity PFA skillset by the wider frontline population, thus achieving the empowerment of the frontline healthcare workforce in dealing with crises at a population level in future emergencies.

## Ethics Statement

This trial has been approved by the Institution Review Board from Central South University (LYG2020029) and by the Psychiatry, Nursing and Midwifery Research Ethics Committee at King's College London, England (LRS/DP-21/22-23161). All participants will be asked to provide their written and informed consent.

## Author Contributions

IN, ML, and LW were responsible for the research concept development. LW drafted the manuscript, IN and ML contributed to the text and critically revised the manuscript. XL contributed to methodology and trial registration. YL, TX, and LW obtained funding and support from the local authorities. All authors approved the final version of the manuscript.

## Funding

It is supported by the health research project from Health Commission of Hunan Province (20190365) and the innovative education project of Central South University (2018CXKZ06).

## Conflict of Interest

The authors declare that the research was conducted in the absence of any commercial or financial relationships that could be construed as a potential conflict of interest.

## Publisher's Note

All claims expressed in this article are solely those of the authors and do not necessarily represent those of their affiliated organizations, or those of the publisher, the editors and the reviewers. Any product that may be evaluated in this article, or claim that may be made by its manufacturer, is not guaranteed or endorsed by the publisher.
