# Supplementary material for: Evaluating a Psychological First Aid Training Intervention (Preparing Me) to Support the Mental Health and Wellbeing of Chinese Healthcare Workers During Healthcare Emergencies: Protocol for a Randomized Controlled Feasibility Trial
Source: Front Psychiatry. 2022 Jan 27;12:809679. doi: 10.3389/fpsyt.2021.809679 (PMC8830777; doi:10.3389/fpsyt.2021.809679)
Supplement: Supplementary file 1 [file Data_Sheet_1.docx]

Supplementary Material

**Knowledge, skills and attitudes questionnaire**

Adapted from Johns Hopkins PFA training evaluation study

Ref: McCabe, O. L., Semon, N. L., Thompson, C. B., Lating, J. M., Everly Jr, G. S., Perry, C. J., ... & Links, J. M. (2014). Building a national model of public mental health preparedness and community resilience: validation of a dual-intervention, systems-based approach. *Disaster medicine and public health preparedness*, *8*(6), 511-526.

A self-report, 5-point Likert-scale composed of 18 items, organized by knowledge (7 items), skills (7 items), and attitudes (3 items) facilitative of stressful events response.

|  | item | Strongly disagree  1 | Disagree  2 | Neutral  3 | Agree  4 | Strongly agree  5 |
| --- | --- | --- | --- | --- | --- | --- |
| Knowledge: self-reported understanding of: | | | | | | |
|  | The concept of stress, crisis |  |  |  |  |  |
|  | The logic of extending PFA training |  |  |  |  |  |
|  | Principles of the stress response & relaxation training |  |  |  |  |  |
|  | Characteristics of acute stress disorder |  |  |  |  |  |
|  | Predictor of PTSD |  |  |  |  |  |
|  | Principles of screening for depression and suicidality, psychosis |  |  |  |  |  |
|  | 5 core components of PFA |  |  |  |  |  |
|  | Important questions to ask before deployment |  |  |  |  |  |
|  | 4 self-care practices for own selves |  |  |  |  |  |
| Skills: perceived self-efficacy, proficiency and ability to: | | | | | | |
|  | Use listening skills to build rapport |  |  |  |  |  |
|  | Discern meanings and feelings from statements |  |  |  |  |  |
|  | Prioritize the needs of a distressed one |  |  |  |  |  |
|  | Differentiate severe from moderate distress |  |  |  |  |  |
|  | Teach/demonstrate diaphragmatic breathing |  |  |  |  |  |
|  | Respond to mental health referral needs |  |  |  |  |  |
|  | Overall PFA self-efficacy |  |  |  |  |  |
| Attitudes: endorsed attitudes, beliefs, and motivations: | | | | | | |
|  | Likelihood of a stress situation |  |  |  |  |  |
|  | Likelihood of stress-caused need for PFA |  |  |  |  |  |
|  | Perceived effectiveness of correctly applied PFA |  |  |  |  |  |
|  | Current adequacy of crisis mental health preparedness |  |  |  |  |  |
|  | Willingness to be a listener and supporter |  |  |  |  |  |

**One month follow-up survey of Psychological First Aid use and usefulness in real-world contexts**

Adapted from Johns Hopkins PFA training evaluation study

Ref: McCabe, O. L., Semon, N. L., Thompson, C. B., Lating, J. M., Everly Jr, G. S., Perry, C. J., ... & Links, J. M. (2014). Building a national model of public mental health preparedness and community resilience: validation of a dual-intervention, systems-based approach. *Disaster medicine and public health preparedness*, *8*(6), 511-526.

The PFA Training Follow-up Questionnaire is an 11-item survey distributed to trainees 1 month after training to determine the frequency (use) and effectiveness (usefulness) of PFA applied to persons who incurred trauma during a disaster event or other personal crisis.

|  | item | Not at all | Frequency of use, once or twice | Three or more times |
| --- | --- | --- | --- | --- |
| Use of PFA |  |  |  |  |
|  | Disaster of other public health crisis |  |  |  |
|  | Non-disaster crisis, other critical incidents in frontline |  |  |  |
| Usefulness of PFA training |  | Disagree or strongly disagree | Agreement with statement, agree or strongly agree | Don’t know |
|  | 1 More willing to provide PFA to survivors of disasters and other critical incidents |  |  |  |
|  | 2 More confident in ability to provide PFA to survivors of disasters and other critical incidents |  |  |  |
|  | 3 Better listener |  |  |  |
|  | 4 Better expressing empathy |  |  |  |
|  | 5 Better at establishing rapport |  |  |  |
|  | 6 Better able to differentiate psychological distress from dysfunction |  |  |  |
|  | 7 More confident in ability to make a referral and serve the roles as liaison and advocate for those in need |  |  |  |
|  | 8 More motivated to participate in general crisis response support |  |  |  |

**Brief resilience scale**

Smith, B.W., Dalen, J., Wiggins, K., Tooley, E., Christopher, P. and Bernard, J. (2008). The Brief Resilience Scale: Assessing the Ability to Bounce Back. ​*International Journal of Behavioral Medicine*​, 15, 194-200.

|  | item | Strongly disagree  1 | Disagree  2 | Neutral  3 | Agree  4 | Strongly agree  5 |
| --- | --- | --- | --- | --- | --- | --- |
| BRS1 | I tend to bounce back quickly after hard times. |  |  |  |  |  |
| BRS2 | I have a hard time making it through stressful events. |  |  |  |  |  |
| BRS3 | It does not take me long to recover from a stressful event. |  |  |  |  |  |
| BRS4 | It is hard for me to snap back when something bad happens. |  |  |  |  |  |
| BRS5 | I usually come through difficult times with little trouble. |  |  |  |  |  |
| BRS6 | I tend to take a long time to get over setbacks in my life. |  |  |  |  |  |

**General self-efficacy scale**

Schwarzer, R., & Jerusalem, M. (1995). Generalized Self-Efficacy scale. In J. Weinman, S. Wright, & M. Johnston, Measures in health psychology: A user’s portfolio. Causal and control beliefs (pp. 35-37). Windsor, UK: NFER-NELSON.

|  | item | Not at all true | Hardly true | Moderately true | Exactly true |
| --- | --- | --- | --- | --- | --- |
| 1 | I can always manage to solve difficult problems if I try hard enough |  |  |  |  |
| 2 | If someone opposes me, I can find the means and ways to get what I want |  |  |  |  |
| 3 | It is easy for me to stick to my aims and accomplish my goals |  |  |  |  |
| 4 | I am confident that I could deal efficiently with unexpected events |  |  |  |  |
| 5 | Thanks to my resourcefulness, I know how to handle unforeseen situations |  |  |  |  |
| 6 | I can solve most problems if I invest the necessary efforts |  |  |  |  |
| 7 | I can remain calm when facing difficulties because I can rely on my coping abilities |  |  |  |  |
| 8 | When I am confronted with a problem, I can usually find several solutions |  |  |  |  |
| 9 | If I am in trouble, I can usually think of a solution |  |  |  |  |
| 10 | I can usually handle whatever comes my way |  |  |  |  |

**The Impact of Event Scale - Revised**

Instructions: below is a list of difficulties people sometimes have after stressful events. Please read each item, and then indicate how distressing each difficulty has been for you during the past seven days with respect to , which occurred on . how much were you distressed or bothered by these difficulties?

Item response anchors are 0=not at all; 1= a little bit; 2= moderately; 3= quite a bit; 4= extremely.

The Intrusion subscale is the MEAN item response of item 1,2,3,6,9,14,16,20. Thus, scores can range from 0 through 4.

The Avoidance subscale is the MEAN item response of items 5,7,8,111,12,13,17,22. Thus, scores can range from 0 through 4.

The Hyperarousal subscale is the MEAN item response of items 4,10,15,18,19,21. Thus, scores can range from 0 through 4.

1. any reminder brought back feelings about it.

2. I had trouble staying asleep.

3. other things kept making me think about it.

4. I felt irritable and angry.

5. I avoided letting myself get upset when I thought about it or was reminded of it.

6. I thought about it when I didn’t mean to.

7. I felt as if it hadn’t happened or wasn’t real.

8. I stayed away from reminders of it.

9. Pictures about it popped into my mind.

10. I was jumpy and easily startled.

11. I tried not to think about it.

12. I was aware that I still had a lot of feelings about it, but I didn’t deal with them.

13. my feelings about it were kind of numb.

14. I found myself acting or feeling like I was back at that time.

15. I had trouble falling asleep.

16. I had waves of strong feelings about it.

17. I tried to remove it from my memory.

18. I had trouble concentrating.

19. reminders of it caused me to have physical reactions, such as sweating, trouble breathing, nausea, or a pounding heart.

20. I had dreams about it.

21. I felt watchful and on-guard.

22. I tried not to talk about it.

**The Simplified Coping Style Questionnaire**

Instructions: How would you describe the way how frequently adopt on each item from Never=0 Occasionally=1 Sometime=2 often=3.

1. Freedom through work, study or some other activities

2. Talk to people and tell your inner troubles

3. Try to see the good side of things

4. Change your mind and rediscover what is important in life

5. Don't take the problem too seriously

6. Stand on your own ground and fight for what you want

7. Find out a few different ways to solve the problem

8. Seek advice from relatives, friends or classmates

9. Change some of the original practices or some of your own problems

10. Learn from others' methods of dealing with similar difficult situations

11. Seek hobbies and actively participate in cultural and sports activities

12. Try to restrain yourself from disappointment, regret, sadness and anger

13. Attempt to take a break or vacation to temporarily put aside the problem (worries)

14. Eliminate worries by smoking, drinking, taking medicine and eating

15. Think that time will change the status quo, the only thing to do is to wait

16. Try to forget the whole thing

17. Rely on others to solve problems

18. Accept the reality, because there is no other way

19. Fantasy that some miracle may happen to change the status quo

20. Comfort yourself

**Post-traumatic Growth Inventory**

Tedeschi, R.G., & Calhoun, L.G. (1996). The Posttraumatic Growth Inventory: Measuring the positive legacy of trauma. ,Journal of Traumatic Stress, 9, 455- 471.

Indicate for each of the statements below the degree to which this change occurred in your life as a result of the crisis/disaster, using the following scale.

0 = I did not experience this change as a result of my crisis.
1 = I experienced this change to a very small degree as a result of my crisis. 2 = I experienced this change to a small degree as a result of my crisis.
3 = I experienced this change to a moderate degree as a result of my crisis. 4 = I experienced this change to a great degree as a result of my crisis.
5 = I experienced this change to a very great degree as a result of my crisis.

|  | item | 0 | 1 | 2 | 3 | 4 | 5 |
| --- | --- | --- | --- | --- | --- | --- | --- |
| 1 | I changed my priorities about what is important in life |  |  |  |  |  |  |
| 2 | I have a greater appreciation for the value of my own life |  |  |  |  |  |  |
| 3 | I developed new interests |  |  |  |  |  |  |
| 4 | I have a greater feeling of self-reliance |  |  |  |  |  |  |
| 5 | I have a better understanding of spiritual matters |  |  |  |  |  |  |
| 6 | I more clearly see that I can count on people in times of trouble |  |  |  |  |  |  |
| 7 | I established a new path for my life |  |  |  |  |  |  |
| 8 | I have a greater sense of closeness with others |  |  |  |  |  |  |
| 9 | I am more willing to express my emotions |  |  |  |  |  |  |
| 10 | I know better that I can handle difficulties |  |  |  |  |  |  |
| 11 | I am able to do better things with my life |  |  |  |  |  |  |
| 12 | I am better able to accept the way things work out |  |  |  |  |  |  |
| 13 | I can better appreciate each day |  |  |  |  |  |  |
| 14 | New opportunities are available which wouldn’t have been otherwise |  |  |  |  |  |  |
| 15 | I have more compassion for others |  |  |  |  |  |  |
| 16 | I put more effort into my relationships |  |  |  |  |  |  |
| 17 | I am more likely to try change things which need changing |  |  |  |  |  |  |
| 18 | I have a stronger religious faith |  |  |  |  |  |  |
| 19 | I discovered that I’m stronger than I thought I was |  |  |  |  |  |  |
| 20 | I learned a great deal about how wonderful people are |  |  |  |  |  |  |
| 21 | I better accept needing others |  |  |  |  |  |  |

**Brief Symptom Inventory - 18**

Instructions: All the questions apply to the two preceding weeks and were to be rated by using “0 = not at all”, “1 = several days”, “2 = more than half the days”, and “3 = nearly every day”.

1 faintness or dizziness

2 feeling no interest in things

3 nervousness or shakiness inside

4 pains in heart or chest

5 feeling lonely

6 feeling tense or keyed up

7 nausea or upset stomach

8 feeling blue

9 suddenly scared for no reason

10 trouble getting your breath

11 feelings of worthlessness

12 spells of terror or panic

13 numbness or tingling in parts of your body

14 feeling hopeless about the future

15 feeling so restless you could not sit still

16 feeling weak in parts of your body

17 thoughts of ending your life

18 feeling fearful

**Professional Quality of Life**

When you *[help]* people you have direct contact with their lives. As you may have found, your compassion for those you *[help]* can affect you in positive and negative ways. Below are some questions about your experiences, both positive and negative, as a *[helper]*. Consider each of the following questions about you and your current work situation. Select the number that honestly reflects how frequently you experienced these things in the *last 30 days*.

**1=never 2=rarely 3=sometimes 4=often 5=very often**

1. I am happy.

2.I am preoccupied with more than one person I *[help]*.

3. I get satisfaction from being able to *[help]* people.

4.I feel connected to others.

5. I jump or am startled by unexpected sounds.

6. I feel invigorated after working with those I *[help]*.

7. I find it difficult to separate my personal life from my life as a *[helper]*.

8. I am not as productive at work because I am losing sleep over traumatic experiences of a person I *[help]*.

9. I think that I might have been affected by the traumatic stress of those I *[help]*.

10. I feel trapped by my job as a *[helper]*.

11. Because of my *[helping]*, I have felt "on edge" about various things.

12. I like my work as a *[helper]*.

13. I feel depressed because of the traumatic experiences of the people I *[help].*

14. I feel as though I am experiencing the trauma of someone I have *[helped]*.

15. I have beliefs that sustain me.

16. I am pleased with how I am able to keep up with *[helping]* techniques and protocols.

17. I am the person I always wanted to be.

18. My work makes me feel satisfied.

19. I feel worn out because of my work as a *[helper].*

20. I have happy thoughts and feelings about those I *[help]* and how I could help them.

21. I feel overwhelmed because my case [work] load seems endless.

22. I believe I can make a difference through my work.

23. I avoid certain activities or situations because they remind me of frightening experiences of the people I *[help]*.

24. I am proud of what I can do to *[help]*.
 25. As a result of my *[helping]*, I have intrusive, frightening thoughts.

26. I feel "bogged down" by the system.
 27. I have thoughts that I am a "success" as a *[helper]*.
 28. I can't recall important parts of my work with trauma victims.
 29. I am a very caring person.
 30. I am happy that I chose to do this work.
